# Effective information spreading based on local information in correlated networks

**DOI:** 10.1038/srep38220

**Published:** 2016-12-02

**Authors:** Lei Gao, Wei Wang, Liming Pan, Ming Tang, Hai-Feng Zhang

**Affiliations:** 1 Web Sciences Center, University of Electronic Science and Technology of China, Chengdu 610054, China; 2 Big Data Research Center, University of Electronic Science and Technology of China, Chengdu 610054, China; 3 School of Mathematical Science, Anhui University, Hefei 230601, China

## Abstract

Using network-based information to facilitate information spreading is an essential task for spreading dynamics in complex networks. Focusing on degree correlated networks, we propose a preferential contact strategy based on the local network structure and local informed density to promote the information spreading. During the spreading process, an informed node will preferentially select a contact target among its neighbors, basing on their degrees or local informed densities. By extensively implementing numerical simulations in synthetic and empirical networks, we find that when only consider the local structure information, the convergence time of information spreading will be remarkably reduced if low-degree neighbors are favored as contact targets. Meanwhile, the minimum convergence time depends non-monotonically on degree-degree correlation, and a moderate correlation coefficient results in the most efficient information spreading. Incorporating the local informed density information into contact strategy, the convergence time of information spreading can be further reduced, and be minimized by an moderately preferential selection.

In the last decade, spreading dynamics in complex networks has attracted much attention from disparate disciplines, including mathematics, physics, social sciences, etc.^1^,^2^,^3^,^4^. Spreads of rumors^5^,^6^,^7^, innovations^8^,^9^,^10^, credits^11^, behaviors^12^ and epidemics^13^,^14^ were studied both in theoretical and empirical aspects. Spreading models, such as susceptible-infected (SI)^15^,^16^,^17^, susceptible-infected-susceptible (SIS)^18^,^19^ and susceptible-infected-recovered (SIR)^20^,^21^,^22^ have been studied to investigate the essential aspects of spreading processes in complex networks^23^. Theoretical studies revealed that underlying network structure have significant impacts on the outbreak threshold as well as outbreak size^3^. Specially, for scale-free networks with degree exponent *γ* ≤ 3, the outbreak threshold vanishes in the thermodynamic limit^18^,^19^,^24^,^25^. Further studies revealed that the degree heterogeneity promotes spreading outbreaks, however limits the outbreak size at large transmission probability^13^.

Utilizing network information to effectively enhance the spreading speed and outbreak size is an important topic in spreading dynamics studies^26^,^27^,^28^,^29^,^30^. The studies on effective information spreading can provide inspiration for epidemic controlling^31^,^32^,^33^, as well as marketing strategies optimization^34^,^35^,^36^. Methods for effective spreading roughly fall into two categories: one is to choose influential nodes as the spreading sources^37^,^38^, while the other is to employ proper contact strategies to optimize spreading paths^39^. Noticeable methods have been proposed for both the two classes. For the identification methods of influential nodes, Kitsak *et al*. revealed that selecting nodes with high *k*-shells as spreading sources can effectively enhance the spreading size^26^. Recently, Morone *et al*. proposed an optimal percolation method to identify the influential nodes^40^. As for the contact process (CP) without bias in heterogenous networks, scholars found that the spreading process follows a precise hierarchical dynamics, i.e., the hubs are firstly informed, and the information pervades the network in a progressive cascade across smaller degree classes^41^. Yang *et al*. proposed a biased contact process by using the local structure information in uncorrelated networks, and their results indicate that the spreading can be greatly enhanced if the small-degree nodes are preferentially selected^39^,^42^. Rumor spreading and random walk models with biased contact strategy were also studied in refs 43 and 44.

Previous results have manifested that in uncorrelated networks, designing a proper contact strategy can effectively promote the information spreading. However, degree-degree correlations (i.e., assortative mixing by degree) are ubiquitous in real world networks^45^,^46^,^47^. A positive degree-degree correlation coefficient indicates that nodes tend to connect to other nodes with similar degrees. While for negative correlation coefficients, large-degree nodes are more likely to connect to small-degree nodes. The degree-degree correlations have significant impacts on spreading dynamics. For instance, assortative (dissortative) networks have a smaller (larger) outbreak threshold, however outbreak size is on the contrary inhibited (promoted) at large transmission rates^48^,^49^.

Although correlations are prevalent in real-world systems, there still lack studies of effective spreading strategy focusing on correlated networks. To promote the information spreading in correlated networks is the motivation of this paper. We propose a preferential contact strategy (PCS), based on the local information of network structure and informed nodes densities. Our findings demonstrate that, when the strategy only consider local structure (LS) information, small-degree nodes should be preferentially contacted to promote the spreading speed, irrespective to the values of degree correlation coefficients. For highly assortative or disassortative networks, small-degree nodes should be more strongly favored to achieve the fastest spreading. Actually, the minimum convergence time of information spreading depends non-monotonically on the correlation coefficient. In addition, we find that the spreading can be further promoted when the information of informed density is incorporated into the PCS. The local informed density (LID) based strategy can better accelerate the spreading, as compared with the global informed density (GID) case.

## Results

### Model of correlated network

To study the interplay of degree correlations and contact strategies, we build correlated networks with adjustable correlation coefficients by employing a degree-preserving edge rewiring procedure. First we generate uncorrelated configuration networks (UCN)^50^ with power-law degree distributions and a targeted mean degree. Then, we adjust the degree correlation coefficient by using the biased degree-preserving edge rewiring procedure^51^. Details about the network generation can be found in the Methods Section.

### Model of information spreading

We consider a contact process (CP) of susceptible-informed (SI)^52^ as the information spreading model. For the SI model, each node can either be in susceptible (S) state or informed (I) state. Initially, a small fraction of nodes are chosen uniformly as informed nodes, while the remainings are in the S state. At each time step, each informed node *i* select *one* of its neighbors *j* to contact with a pre-defined contact probability *W*_*ij*_. If the node *j* is in S state, then it will become I state with the transmission probability *λ*. During the spreading process, the synchronous updating rule is applied, i.e., all nodes will update their states synchronously in each time step^53^. Repeat this process till all nodes are informed, and the network converges to an unique all-informed state. Thus for the model we consider, spreading efficiency can be evaluated by the convergence time *T*, which is defined as the number of time steps that all nodes become informed.

### Preferential contact strategy based on local information

In real spreading processes, it is hard for nodes to known explicitly the states of neighbors. The lack of information may arise many redundant contacts between the two informed nodes in the CP, which will greatly reduce the spreading efficiency. Thus, we propose a preferential contact strategy (PCS), which combines the local structure (LS) and local informed density (LID) information in a comprehensive way. The probability *W*_*ij*_ that an informed node *i* selecting a neighbor *j* for contact is given by


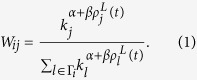


Here Γ_*i*_ is the set of neighbors of *i* and *k*_*i*_ its degree. In addition, *α* and *β* are two tunable parameters. The preferential structure exponent *α* determines the tendency to contact small-degree or large-degree nodes. Large-degree neighbors are preferentially contacted when *α* > 0, while small-degree neighbors are favored when *α* < 0. When *α* = 0 all neighbors are randomly chosen, and it reduces to the classical CP process^52^. The preferential dynamic exponent *β* reflects whether the neighbors with small or large LIDs are favored. For a specific node *j*, the LID is defined as


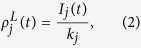


where *I*_*j*_(*t*) is the number of informed neighbors of node *j* at time *t*.

Taking 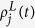 into the contact strategy is based on several reasons. Firstly, suppose there are two neighbors with the same degree, clearly the neighbor with a higher 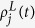 has a larger probability to be already informed. It is reasonable to preferentially choose the neighbor of the smaller 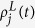 as contact target by setting a suitable negative *β*. Secondly, contacting neighbors with low LIDs can further provide more latent chances to inform the next-nearest neighbors. Third, the LID is relatively easier to obtain, as compared with the global informed density (GID) of network *ρ*^*G*^(*t*) = *I*(*t*)/*N*, where *I*(*t*) is the total number of informed nodes in the network at time *t*. For comparison, we also investigate the performance of GID information based strategy, where the contact probability is given by replacing 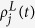 with *ρ*^*G*^(*t*) in Eq. (1).

### Case of *α* = *β* = 0 in uncorrelated networks

**Figure 1:**
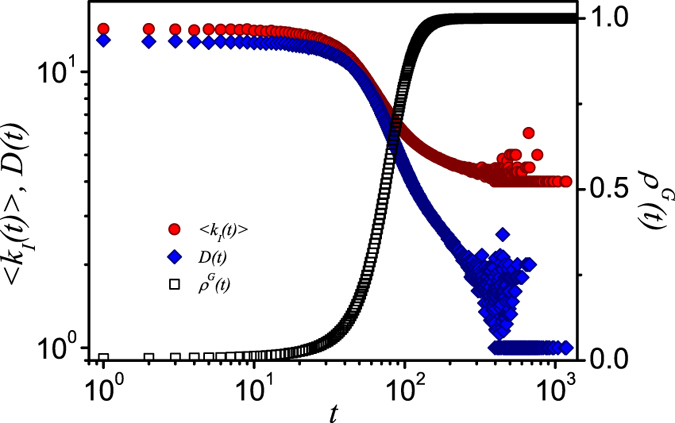
For unbiased contacts, the time evolutions of information spreading in random scale-free networks. The mean degree 〈*k*_*I*_(*t*)〉 of newly informed nodes (red circles), the density of informed nodes *ρ*^*G*^(*t*) (black hollow squares), and the informed diversity of degrees *D*(*t*) (blue diamonds) versus time steps *t*. Other parameters are set as *N* = 10^4^, *γ* = 3.0, 〈*k*〉 = 8, *λ* = 0.1, and 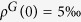 respectively.

We investigate the time evolutions of information spreading with unbiased contact strategy in heterogeneous random networks. When *α* = *β* = 0, hubs have a higher probability to be contacted since they have more neighbors. As a result, the hubs will become informed quickly. In contrast, small-degree nodes with fewer neighbors are less likely to be contacted and informed. To be concrete, the above scenario is illustrated in Fig. 1. We show the time evolutions of the informed density *ρ*^*G*^(*t*), mean degree of newly informed nodes 〈*k*_*I*_(*t*)〉, and the degree diversity of the newly informed nodes *D*(*t*)^54^. Here *D*(*t*) is defined as


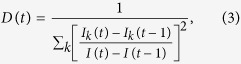


where *I*_*k*_(*t*) is the number of informed nodes with degree *k* at time *t*. The larger values of degree diversity *D*(*t*) indicate that the newly informed nodes are from diverse degree classes.

At initial time steps, the informed density *ρ*^*G*^(*t*) is small, and a large value of 〈*k*_*I*_(*t*)〉 indicates that hubs are quickly informed. The value of *D*(*t*) is also very large during this stage since most nodes are in S state and nodes from all degree classes can be get informed. With the rapid increase of *ρ*^*G*^(*t*), both 〈*k*_*I*_(*t*)〉 and *D*(*t*) decreases, which indicates intermediate degree nodes are gradually informed. In the late stage of spreading, small values of 〈*k*_*I*_(*t*)〉 and *D*(*t*) reveal that the time-consuming part of the spreading is to reach some small-degree nodes. Previous studies showed that the optimal biased contact strategy basing on the neighbor degrees is which when *α* ≈ −1^52^. In this case, the small-degree nodes will be informed more easily, while the central role of the hubs for transmitting information is not excessively weakened. Therefore, balancing the contacts to small-degree and large-degree nodes is essential to the problem of facilitating the spreading.

Assortative and disassortative networks display distinct structure characteristics, with small-degree nodes play different roles^23^. For assortative networks, many small-degree nodes locate in the periphery of the network. While for disassortative networks, some small-degree nodes act as bridges of connecting two large-degree nodes, and with more small-degree nodes act as leaf nodes in the star-like structures (see illustration in Fig. S1 of Supporting Information). While the locations of small degree nodes have been altered by the degree correlations, transmitting information effectively to small degree nodes is essential for facilitating the spreading as discussed above. This suggests that we should treat small degree nodes more carefully in correlated networks. In the LS based PCS, nodes are identified by their degrees, and all neighbors with the same degree are treated as equivalent. This motivates that we could further distinguish the small degree nodes to better enhance the spreading. To this end, we incorporate the LIDs of neighbors into the PCS and favor transmitting information to low informed regions.

### Cases of *β* = 0 in correlated networks

We first study the effect of PCS by only considering the LS information, i.e., *β* = 0. We verify the performance of the contact strategies in scale-free networks with given mean degrees and degree correlation coefficients. The networks are generated according to the method described in the Model Section. The size of networks is set to *N* = 10^4^ and average degree 〈*k*〉 = 8. In addition, we apply the method to two empirical networks which are the Router^55^ and CA-Hep^56^. Initially, 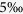 nodes are randomly chosen as the seeds for spreading. Without lose of generality, the transmission probability is set as *λ* = 0.1. All the results are obtained with averaging over 100 different network realizations, with 100 independent runs on each realization.

**Figure 2:**
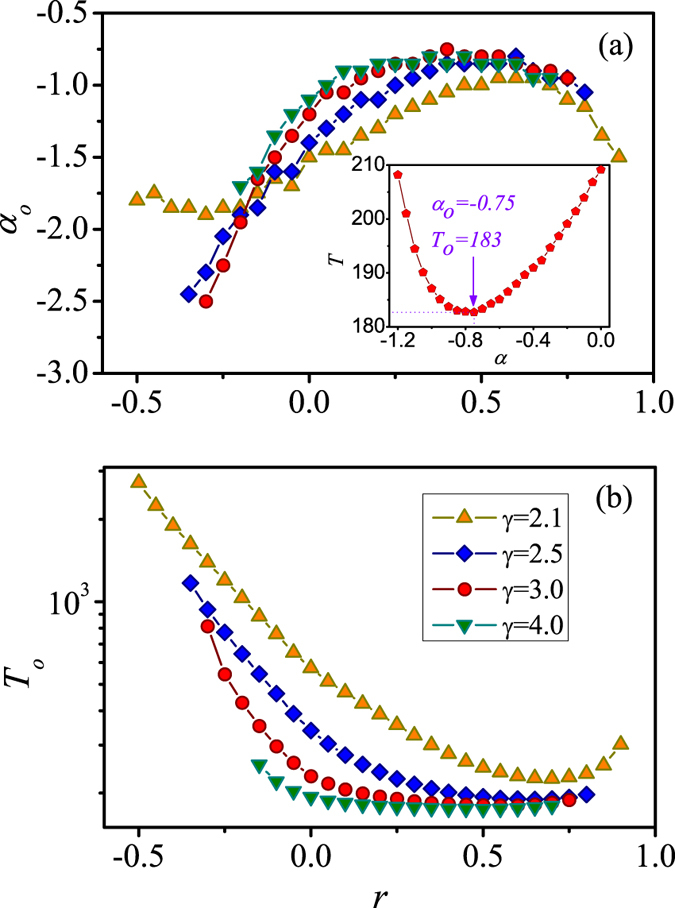
The optimal performance of LS information based PCS in correlated configuration networks. (**a**) The optimal value of preferential structure exponent *α*_*o*_ and (**b**) the convergence time *T*_*o*_ versus correlation coefficient *r* for degree exponents *γ* = 2.1 (orange up triangles), *γ* = 2.5 (blue diamonds), *γ* = 3.0 (red circles), and *γ* = 4.0 (green down triangles), respectively. The inset of (**a**) shows the relationship between *T* and *α* when *γ* = 3.0 and *r* = 0.4. We set other parameters as *N* = 10^4^, 〈*k*〉 = 8, and *λ* = 0.1, respectively.

For a specific network, there always exists an optimal value of preferential structure exponent *α*_*o*_, which will lead to the minimum convergence time *T*_*o*_ [see the inset of Fig. 2(a)]. Figure 2 shows *α*_*o*_, *T*_*o*_ versus *r* for networks with different degree exponent *γ*. From Fig. 2(a), we find that the values of *α*_*o*_ are negative irrespective of *r*. In other words, preferentially contacting small-degree neighbors will promote the spreading efficiency, which is consist with the previous studies for uncorrelated networks^52^. More importantly, *α*_*o*_ depends non-monotonically on *r*. In particular, when *r* is either very large or small, *α*_*o*_ tends to be smaller than that for the intermediate values of *r*. Thus, for highly assortative and disassortative networks, it requires stronger tendency to contact small-degree nodes to achieve optimal spreading. This is due to the distinct local structure characteristics of small degree nodes (see details in Sec. S1 of Supporting Information). We test the method for networks with different values of degree exponent *γ* in Fig. 2(a). It can be seen that for different *γ* the behaviors are similar. However, one noticeable difference is that for *γ* = 2.1, *α*_*o*_ is significantly larger than the other three cases when *r* is small. We argue that this anomaly is caused by the structural constrains imposed by the strong heterogeneity of degrees for *γ* = 2.1. Some structural properties for *γ* = 2.1 and *γ* = 3.0 are summarized in Table S1 (see details in Sec. S2 of Supporting Information). To further clarify the effects of correlation coefficient *r* on the convergence time *T*, we plot the minimum convergence time *T*_*o*_ as a function of *r* in Fig. 2(b). One can see that *T*_*o*_ also depends non-monotonically on *r*. Specifically, *T*_*o*_ first decreases with *r* and then increases. For those highly disassortative networks, many small clusters are interconnected via some small-degree nodes. The inter-cluster transmissions of information delay the spreading and lead to a large value of *T*_*o*_. When *r* is very large for assortative networks, though the core composed of large-degree nodes is easily informed, small nodes in the periphery are harder to be contacted. The core-periphery structure also gives rise to a slightly large value of *T*_*o*_.

**Figure 3:**
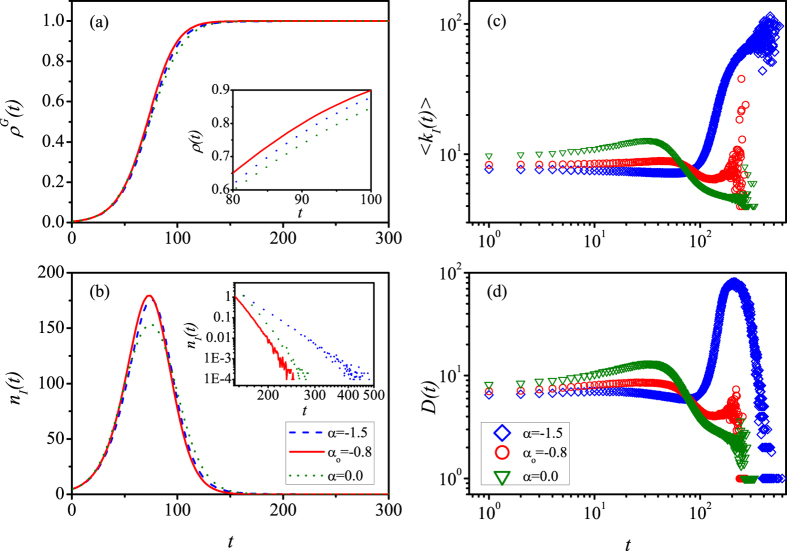
The effect of LS information based PCSs on the time evolution of information spreading in correlated networks. (**a**) The informed density *ρ*^*G*^(*t*), and (**b**) the number *n*_*I*_(*t*), (**c**) mean degree 〈*k*_*I*_(*t*)〉, and (**d**) degree diversity *D*(*t*) of newly informed nodes versus *t* for different values of *α*. Different colors indicate different values of *α*. The inset of (**a**) shows the time evolution of *ρ*^*G*^(*t*) in the time interval [80, 100]. The inset of (**b**) shows *n*_*I*_(*t*) in the time interval [150, 500]. We set other parameters as *N* = 10^4^, *γ* = 3.0, 〈*k*〉 = 8, *λ* = 0.1, and *r* = 0.55, respectively.

To complete the above discussions, we study the time evolution properties of the spreading process. Figure 3(a) depicts the informed density *ρ*^*G*^(*t*) versus time *t* for the case of *r* = 0.55 and *γ* = 3.0. Note that *r* = 0.55 minimize *T*_*o*_ when *γ* = 3, as shown in Fig. 2(b). The three different lines correspond to different values of *α*, which are *α* = −1.5, −0.8, 0.0, respectively. It’s clear from Fig. 3(a) that the case for *α*_*o*_ = −0.8 spreads faster than the two other cases. The number of newly informed nodes *n*_*I*_(*t*) as a function of *t* is given in Fig. 3(b). One can observe that, compared with *α* = 0 and *α* = −1.5, the *n*_*I*_(*t*) for *α*_*o*_ = −0.8 is larger (smaller) than the other two cases at the early (late) stages, indicating the fastest spread of information. When the network is almost fully informed at late stages, the inset in Fig. 3(b) demonstrates that *n*_*I*_(*t*) decays faster with time for *α*_*o*_ = −0.8. Figure 3(c) and (d) respectively show the time evolutions of mean degree of new informed nodes 〈*k*_*I*_(*t*)〉 and the corresponding degree diversity *D*(*t*). For *α*_*o*_ = −0.8, the 〈*k*_*I*_(*t*)〉 and *D*(*t*) remain relatively stable with time. In other words, nodes with different degrees almost have uniform probabilities of being informed, which is close to the ideal situation for effective spreading^52^. However, for *α* = 0 large-degree nodes are first informed and then the small-degree ones, while for *α* = −1.5 the order is reversed. For the two cases, the degree diversity becomes small at the late stages of information spreading. Together with the 〈*k*_*I*_(*t*)〉 we can conclude that the spreading is delayed by small-degree (large-degree) nodes for *α* = 0 (*α* = −1.5). Correspondingly, the results of informed degree diversity *D*(*t*) in Fig. 3(d) validate the advantage of *α*_*o*_ = −0.8 again, which is more stable than that for *α* = 0 and *α* = −1.5.

**Figure 4:**
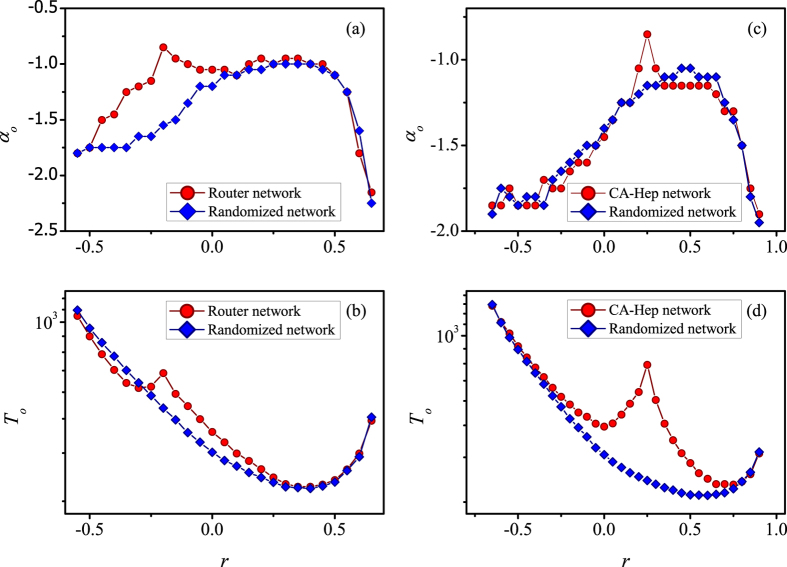
The optimal performance of LS information based PCS in correlated networks by rewiring real-world networks (red circles) and randomized networks (blue diamonds). The optimal value of preferential structure exponent *α*_*o*_ (**a**) and convergence time *T*_*o*_ (**b**) versus correlation coefficient *r* for the Router network. The *α*_*o*_ (**c**) and *T*_*o*_ (**d**) versus *r* for the CA-Hep network.

We also apply the LS information based PCS to two empirical networks. (1) Router. The router level topology of the Internet, collected by the Rocketfuel Project^55^. (2) CA-Hep. Giant connected component of collaboration network of arxiv in high-energy physics theory^56^. More details about the two networks can be found in Sec. S3 of Supporting Information. We find the similar phenomena that observed in Fig. 2, i.e., the non-monotonic dependence of *α*_*o*_ and *T*_*o*_ on *r* for the case of the empirical networks (see Fig. 4). Nevertheless, some abnormal bulges of *α*_*o*_ and *T*_*o*_ emerge at certain values of *r*. By analyzing the structures of the networks, we find that the networks are very similar to the original networks as there are few rewiring edges in the networks at these certain values of *r* with abnormal bulges. Owing to the structural complexity of the real networks, which are significantly different from the synthetic networks, leading to abnormal bulges at certain values of *r*. To prove that the above abnormal phenomenon comes from the structural complexity of the empirical networks, we randomize the empirical networks by sufficient rewiring process but do not change the original degree distribution and the degree of each node. After sufficient times of randomization, we then check the contact strategy in the randomized networks, and one can see the abnormal bulges disappear. Moreover, the curves become more smooth and the non-monotonic phenomenon becomes more evident.

### Cases of *α* = *α*_*o*_ and *β* < 0 in correlated networks

**Figure 5:**
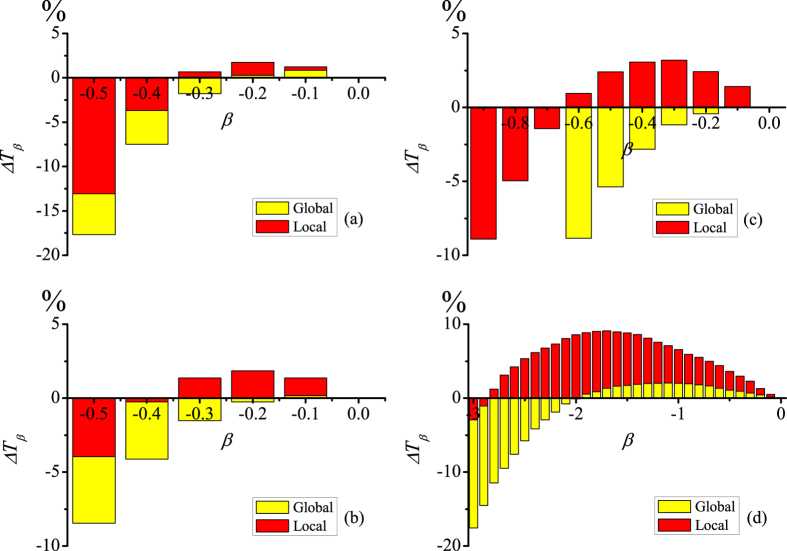
In correlated networks, the effects of LID and GID based PCSs on the convergence time. The relative ratio of the convergence time Δ*T*_*β*_ versus *β* for different *r* values: (**a**) *r* = 0, *α*_*o*_ = −1.2, (**b**) *r* = 0.55, *α*_*o*_ = −0.8, (**c**) *r* = 0.75, *α*_*o*_ = −0.95, and (**d**) *r* = −0.3, *α*_*o*_ = −2.5, respectively. We set other parameters as *N* = 10^4^, *γ* = 3.0, 〈*k*〉 = 8, and *λ* = 0.1. It is noted that the results for the GID case are not shown when 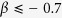 in subfigure (**c**), because their values exceed the minimum value of vertical coordinate. Obviously, Δ*T*_*β*_ monotonically increases with *β*.

When *α* = *α*_*o*_, the LS information based PCS can effectively enhance the spreading efficiency. We further incorporate the LID information, i.e., with *β* < 0 and *α* = *α*_*o*_ in Eq. (1). The spreading efficiency Δ*T*_*β*_ is measured by 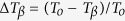, where *T*_*β*_ represents the convergence time when *β* < 0, and *T*_*o*_ denotes the convergence time when *β* = 0. Thus Δ*T*_*β*_ > 0 (Δ*T*_*β*_ < 0) indicates that introducing LID information can enhance (inhibit) the spreading efficiency. For comparison we also study the effects of GID information, by replacing 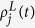 with *ρ*^*G*^(*t*) in Eq. (1). The results in Fig. 5(a), (b) and (c) manifest that, the effective use of LID information can further reduce the convergence time when *β* is set as a small negative value (e.g., *β* = −0.1 and −0.2). Yet, *β* with larger magnitude (e.g., *β* = −0.5) will increase the convergence time. For different values of degree correlation coefficient *r*, there is obviously an optimal value *β*_*o*_ at which the information spreading can be effectively enhanced. Moreover, compared with the GID information, utilizing the LID information not only speeds up the spreading more significantly but also has a wider range of *β* with Δ*T*_*β*_ > 0. For disassortative networks, as shown in Fig. 5(d) with *r* = −0.3, the LID based PCS can speed up the spreading of information for a wide range of *β*. Such an improvement is more evident as compared to uncorrelated [Fig. 5(a)] and assortative networks [Fig. 5(b) and (c)]. We conclude that LID based PCS performs better than the GID case in reducing the convergence time.

**Figure 6:**
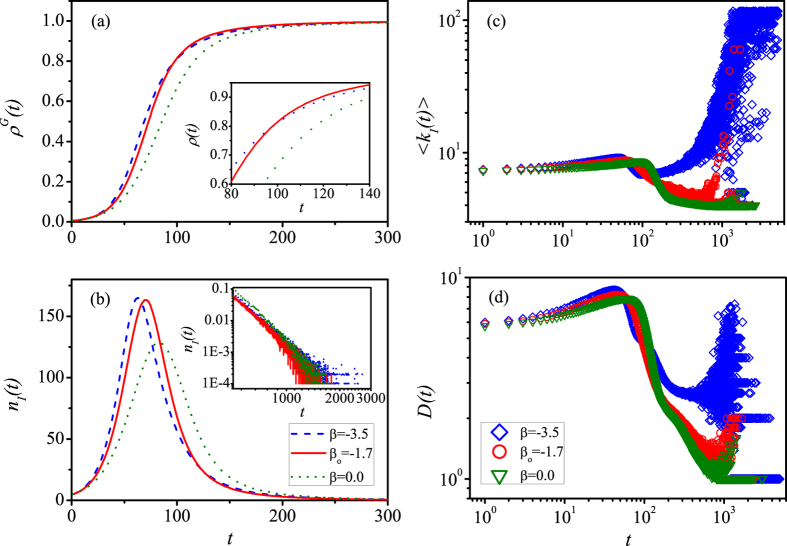
The effect of LID based PCS on the time evolution of information spreading in disassortative networks. (**a**) The informed density *ρ*^*G*^(*t*), and the number *n*_*I*_(*t*) (**b**), mean degree 〈*k*_*I*_(*t*)〉 (**c**) and degree diversity *D*(*t*) (**d**) of newly informed nodes versus *t* for different values of *β*. Different colors indicate different values of *β*. The inset of (**a**) shows the time evolution of *ρ*^*G*^(*t*) in the time interval [80, 140]. The inset of (**b**) shows *n*_*I*_(*t*) in the time interval [550, 3000]. We set other parameters as *γ* = 3.0, *N* = 10^4^, 〈*k*〉 = 8, *λ* = 0.1, *r* = −0.3, and *α* = −2.5, respectively.

Figure 6 presents time evolutions of some statistics of the spreading process in disassortative networks with *r* = −0.3. Figure 6(a) and the inset suggest that the optimal value *β*_*o*_ = −1.7 can better improve the speed of spreading. Figure 6(b) emphasizes that, for the case of *β* < 0, the number of newly informed nodes *n*_*I*_(*t*) increases faster than the case of *β* = 0 at the initial stage. However, the inset of Fig. 6(b) illustrates that, for the case of *β*_*o*_ =−1.7, *n*_*I*_(*t*) goes to zero faster than the case of *β* = −3.5. Similar to Fig. 3(c) and (d), the results in Fig. 6(c) and (d) also manifest that too large (small) values of *β* make the small-degree (large-degree) nodes uneasy to be informed, which will inhibit the spreading. In strongly disassortative networks, complex local structures and dynamical correlations cause nodes with the same degree to be in different local dynamical statuses. The LS information based PCS can not effectively reflect and overcome the difference of local dynamical status. The optimal value *β*_*o*_ guarantee the probability of being informed more homogeneous and steady for different degree classes, leading to the fastest spreading of information. Similarly, we also explain why the PCS based on the LID information yields better performance than the GID case (see details in Sec. S4 of Supporting Information).

Finally, we verify the effectiveness of the informed density information based strategies in Router network and CA-Hep network. Figures S3–S7 show that the LID case performs better in improving the speed of information diffusion, and there exists an optimal value of *β* (see details in Sec. S5 of Supporting Information).

## Conclusions

To effectively promote the information spreading in correlated networks, we proposed a PCS by considering both the LS and LID information. Based on extensive simulations in artificial and real-world networks, we verified the effectiveness of the proposed strategy, and generally found that preferentially selecting nodes with smaller degrees and lower LIDs is more likely to promote information spreading in a given network. First, we studied the strategy which only considers the LS information. For a given network, there generally exists an optimal preferential exponent *α*_*o*_, at which the small-degree nodes are favored and the convergence time *T*_*o*_ reaches its minimum value. Especially, the small-degree nodes should be favored more strongly to achieve optimal spreading when networks are highly assortative or disassortative. Also, the optimal convergence time *T*_*o*_ depends non-monotonically on the correlation coefficient *r*. Then, we induced the informed density into the LS information based PCS with optimal exponent *α*_*o*_. Compared to the strategy with GID, the LID information based PCS reduces the convergence time more significantly. For the LID case, an optimal value *β*_*o*_ can be observed and minimize the convergence time. We further discussed the effects of different parameter combinations (*α, β*) and source nodes on the effectiveness of our proposed PCS based on LID information (see details in Sec. S6 of Supporting Information). We found that these factors do not qualitatively affect the above stated results, i.e., the LID based PCS can effectively accelerate the spreading.

Utilizing network information to improve the spreading is an important topic in spreading dynamics studies. In this work, we study the effect of correlated networks on the effective contact strategy basing on the local structure and informed density. Our results would stimulate further works about contact strategy in the more realistic situation of networks such as community networks^57^,^58^, weighted networks^13^,^59^, temporal networks^60^,^61^, and multiplex networks^33^,^62^. And this work maybe provide reference for the promotion of social contagions such as technical innovations, healthy behaviors, and new products^10^,^11^,^12^. The results presented in our work are based on extensive numerical simulations, how to get accurate theoretical results needs further study. We may get some meaningful insights of the theoretical approaches from other dynamics on complex networks, especially the similar dynamics zero range process^63^,^64^,^65^,^66^,^67^,^68^,^69^, in which a jumping-rate with biasing low-degree nodes would possibly avoid particle condensation.

## Methods

### Uncorrelated configuration model

We generate uncorrelated configuration networks (UCN)^50^ with power-law degree distributions and targeted mean degrees as follows: (1) A degree sequence of *N* nodes is drawn from the power-law distribution *P*(*k*) ~ *k*^−*γ*^, with all the degrees confined to the region 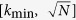, where *γ* is the degree exponent. Note that the average of the degrees is un-controlled but depends on *γ*. (2) Adjust the average of the degree sequence to a targeted value to eliminate the difference of mean degree between synthetic networks with different degree exponents^70^. In detail, to transform mean degree from original mean degree 〈*k*〉_now_ to targeted mean degree 〈*k*〉_tar_, the degree of each node *i* is re-scaled as 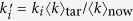. Now the new degrees 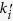 may be not integers, therefore we need to convert then to integers while preserving the degree distribution and the mean degree. Since 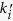 can be written as 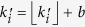 with *b* ∈ [0, 1), we take 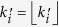 with probability 1 − *b*, while 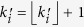 with probability *b*. (3) The nodes with updated degrees are randomly connected via standard procedure of the UCN model.

### Adjusting degree correlation coefficient

We use the biased degree-preserving edge rewiring procedure to adjust the degree correlation coefficient^51^. Note that this procedure is also applicable to empirical networks. The procedure is as follows: (1) At each step, two edges of the network are randomly chosen and disconnected. (2) Then we place another two edges among the four attached nodes, according to their degrees. To generate assortative (dissortaive) networks, the highest degree node is connected to the second highest (lowest) degree node, and also connect the rest pair of nodes. If one or both of these new edges are already exist in the network, the step will be discarded and a new pair of edges will be randomly chosen. (3) Repeat this procedure till the degree correlation coefficient reaches the target value. Here the degree correlation coefficient^23^ is defined as:


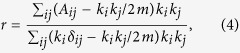


where *m* is the total number of edges in the network, *A* is the adjacency matrix (If there is an edge between nodes i and j, *A*_*i*_*j* = 1; otherwise, *A*_*i*_*j* = 0.) and *δ*_*ij*_ is the Kronecker delta (which is 1 if *i* = *j* and 0 otherwise.). When *r* = 0 there is no degree-degree correlation in the network, while *r* > 0 and *r* < 0 indicate positive and negative degree-degree correlations respectively.

## Additional Information

**How to cite this article**: Gao, L. *et al*. Effective information spreading based on local information in correlated networks. *Sci. Rep.* **6**, 38220; doi: 10.1038/srep38220 (2016).

**Publisher's note:** Springer Nature remains neutral with regard to jurisdictional claims in published maps and institutional affiliations.

## Supplementary Material

Supplementary Information
