# Supplementary material for: Effective information spreading based on local information in correlated networks
Source: Sci Rep. 2016 Dec 2;6:38220. doi: 10.1038/srep38220 (PMC5133588; doi:10.1038/srep38220)
Supplement: Supplementary Information [file srep38220-s1.pdf]

Supporting Information for

Effective information spreading based on local information in correlated networks

Lei Gao, Wei Wang, Liming Pan, Ming Tang,and Hai-Feng Zhang

Contents

S1. Distinct locations of small degree nodes in disassortative and assortative networks 2
S2. The comparision of structural properties for disassortative networks with $\gamma = 2.1$ and $\gamma = 3.0$ 2
S3. Structural properties of the empirical networks 3
S4. Time evolutions of information spreading for the LID and GID cases 3
S5. Cases of $\alpha = \alpha_o$ and $\beta < 0$ in empirical networks 3
S6. The LID based PCS with general parameter combinations (α, β) 4
References 5

S1. Distinct locations of small degree nodes in disassortative and assortative networks

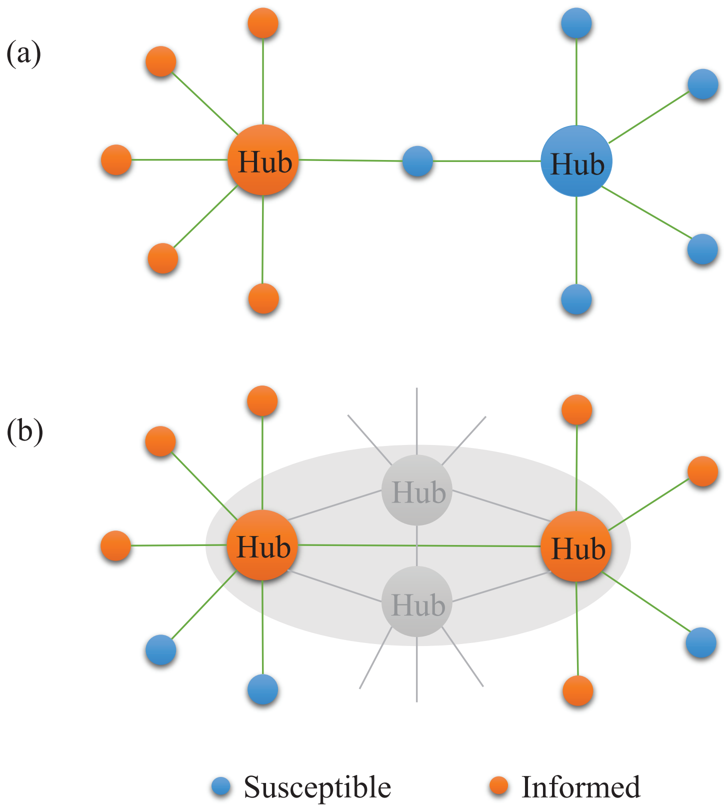

FIG. S1: **Illustration of SI model in the CP for (a) disassortative networks and (b) assortative networks.** Each node can be in S (blue) state or I (orange) state.

In extremely disassortative networks, hubs are surrounded by small-degree nodes and thus form star-like structures. Usually the hubs are not directly connect to each other and the star-like groups are interconnected via small-degree nodes. A typical such structure is illustrated in Fig. S1(a). To quickly transmit the information from an informed star-like group to other groups, the small-degree nodes, which take the role of bridges, demand to be more preferentially contacted. For the extremely assortative networks, large-degree nodes form a rich club and locate in the core of networks, while small-degree nodes locate in the periphery, as shown in Fig. S1(b). In this case, the information can easily spread in the core. But small-degree nodes should be preferentially contacted to avoid redundant contacts among hubs. In conclusion, both for highly assortative and disassortative networks, small-degree nodes should be more favored. When tuning the correlation coefficient of network, say from assortative to disassortative, the core-periphery structures gradually break up and turn into the star-like structures. During this process, α_o first increases and then decreases. This explains the non-monotonic relationship between r and α_o.

S2. The comparision of structural properties for disassortative networks with $\gamma = 2.1$ and $\gamma = 3.0$

Some structural properties for $\gamma = 2.1$ and $\gamma = 3.0$ are summarized in table S1. For $\gamma = 3.0$ and $r = -0.3$, the mean degree of neighbors of the highest-degree node $\langle k \rangle_{\Gamma(\mathrm{h})}$ is small and close to the minimum degree of the network $k_{\min}$. In addition, we measure the degree heterogeneity of neighboring nodes of the highest-degree node $H_{\Gamma(\mathrm{h})}$. Low value

of $H_{\Gamma(\mathrm{h})}$ indicates that almost all the neighbors have very small degrees, which further implies the star-like structure around the hubs. On the contrary, for $\gamma = 2.1$, $\langle k\rangle_{\Gamma(\mathrm{h})}$ is much larger than $k_{\min}$ and also the $H_{\Gamma(\mathrm{h})}$ is of larger values. That is to say, the star-like structure around hubs is less significant for $\gamma = 2.1$. In this case, small nodes do require too strong bias to achieve optimal spreading. This explains the anomaly of α_o for $\gamma = 2.1$ and $r = -0.3$. For smaller values of $r = -0.4, r = -0.5$, $\langle k\rangle_{\Gamma(\mathrm{h})}$ and $H_{\Gamma(\mathrm{h})}$ also become smaller there is no very obvious star-like structure, and the α_o thus remains unchanged.

TABLE S1: **Some statistics of network properties for different degree exponents.** Structural properties include the mean minimum degree $k_{\min}$ of networks, mean degree $\langle k_{\max}\rangle$, mean neighboring degree $\langle k\rangle_{\Gamma(\mathrm{h})}$ and neighboring degree heterogeneity $H_{\Gamma(\mathrm{h})}$ of the largest-degree nodes. The neighboring degree heterogeneity is defined as $H_{\Gamma(\mathrm{h})} = \langle k^2\rangle_{\Gamma(\mathrm{h})}/\langle k\rangle^2_{\Gamma(\mathrm{h})}$, where $\langle k\rangle_{\Gamma(\mathrm{h})}$ and $\langle k^2\rangle_{\Gamma(\mathrm{h})}$ are the first and second moments of neighboring degrees, respectively.

γ	r	$k_{\min}$	$\langle k_{\min}\rangle$	$\langle k\rangle_{\Gamma(\mathrm{h})}$	$H_{\Gamma(\mathrm{h})}$
3	-0.3	4	112.5	4.4	1.031
2.1	-0.3	2	134.5	10.3	4.357
2.1	-0.4	2	134.5	5.4	4.024
2.1	-0.5	2	134.5	3.1	2.366

S3. Structural properties of the empirical networks

We wish to investigate how the correlation coefficients r affect the optimal value of preferential structure exponent. This is achieved by rewiring the original network with preserving the degree sequence. However, due to the abundance of degree 1 nodes in the two empirical networks, the correlation coefficients are confined to a small region. Also, with those degree 1 nodes it is difficult to adjust the correlation coefficients while preserving the connectivity of networks. To overcome this problem, we remove all 1-shell nodes from the original networks [1]. Briefly, first we remove all the nodes with degree 1, and then re-calculate the degrees of nodes. This procedure is repeated until the degrees of all nodes are greater than one. To exhibit the structural complexity of the empirical networks, we randomize the empirical networks by sufficient rewiring process but do not change the original degree distribution and the degree of each node. Some structural properties of the two networks (after removing 1-shell nodes) are presented in Table S2.

TABLE S2: **Structural properties of the empirical networks considered in this paper.** Structural properties include number of nodes N, number of edges E, mean degree $\langle k\rangle$, maximum degree $k_{\max}$, degree heterogeneity $H_k = \langle k^2\rangle/\langle k\rangle^2$, average shortest distance L, correlation coefficient r, and clustering coefficient C [2, 3].

Data		N	E	$\langle k\rangle$	$k_{\max}$	H_k	L	r	C
Router	Real	728	1964	5.4	59	2.537	5.232	-0.216	0.168
	Randomized	728	1964	5.4	59	2.537	3.583	-0.047	0.040
CA-Hep	Real	7059	23227	6.6	65	2.001	5.656	0.247	0.613
	Randomized	7059	23227	6.6	65	2.001	4.386	-0.004	0.003

S4. Time evolutions of information spreading for the LID and GID cases

The time evolutions of $\rho^G(t)$ [Fig. S2(a)] and $n_I(t)$ [Fig. S2(b)] show that the spreading speed for the LID case is quicker than that for the GID case. Since the local density information can better reveal the information distribution in a local region, some small-degree nodes with low informed density of neighbors can be informed early [see Figs. S2(c) and (d)]. As a result, the information can diffused to whole networks more effectively as compared to the GID case.

S5. Cases of $\alpha = \alpha_o$ and $\beta < 0$ in empirical networks

We verify the effectiveness of the informed density information based strategies in Router network and CA-Hep network. All the real (Figs. S3-S5) and correlated (Figs. S6-S7) empirical networks show the similar results with artificial networks. On one hand, the convergence time can be reduced when β is slightly below zero for the local density strategy. Nevertheless, too small values of β will instead increase the convergence time. In other words, there

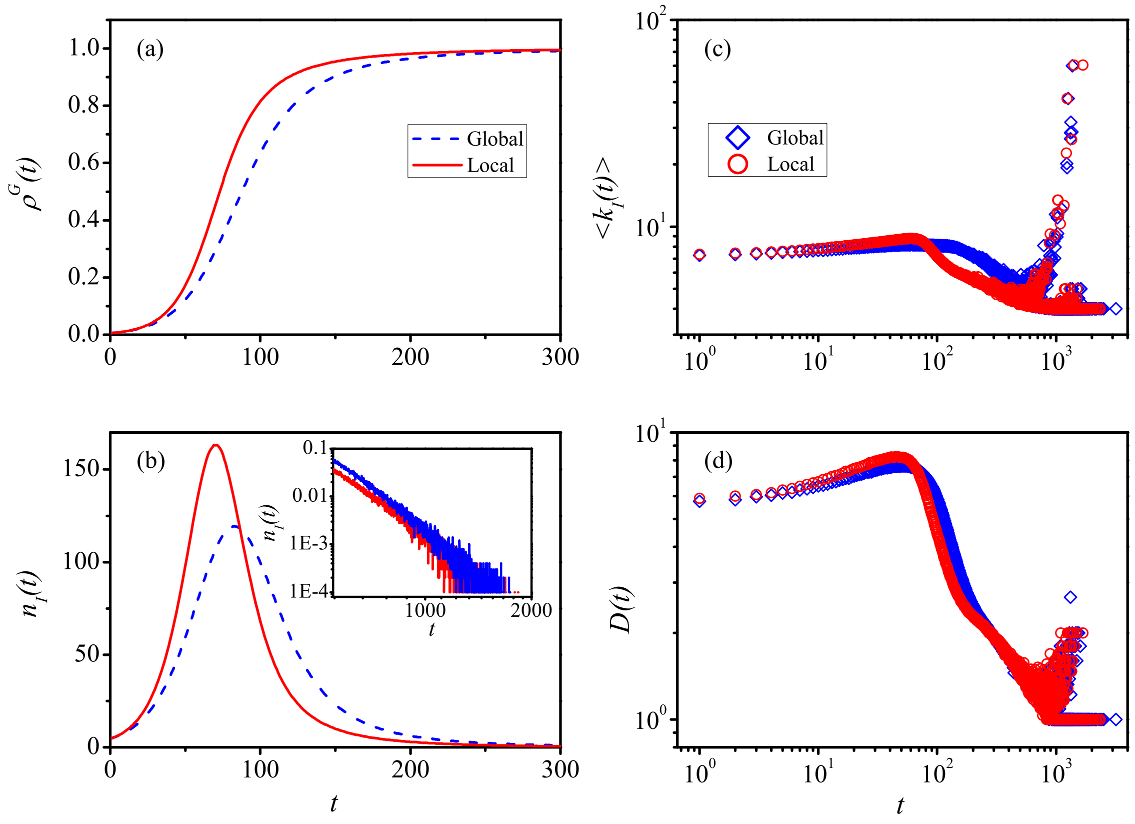

FIG. S2: **Comparison of the effects of LID and GID based PCSs on the time evolution of information spreading in disassortative networks.** (a) The informed density $\rho^G(t)$, and the number $n_I(t)$ (b), mean degree $\langle k_I(t)\rangle$ (c) and degree diversity $D(t)$ (d) of newly informed nodes versus t for different values of β by using GID (color blue) or LID (color red). The inset of (b) shows $n_I(t)$ in the time interval $[550, 2000]$. We set other parameters as $\gamma = 3.0$, $N = 10^4$, $\langle k\rangle = 8$, $\lambda = 0.1$, $r = -0.3$, $\alpha = -2.5$, and $\beta = -1.7$ respectively.

is an optimal value β_o at which the information spreading can be effectively enhanced. On the other hand, introducing the local density information not only reduces the convergence time more significantly, but also yields a wider region of effective β as compared with the global case. Thus, the local local density based contact strategy performs better in improving the speed of information diffusion.

S6. The LID based PCS with general parameter combinations (α, β)

We use heat maps to reveal the dependence of the convergence time on different parameter combinations (α, β) in uncorrelated networks (see Fig. S8) and assortative networks (see Fig. S9). Two source selecting strategies are compared: randomly chosen source nodes in Figs. S8(a) and S9(a), and in Figs. S8(b) and S9(b) the largest-degree nodes are selected greedily as sources. We found that, in different network structures, different parameters (α, β) and source nodes can not qualitatively affect the results shown in the main text, i.e., the LID based PCS can effectively accelerate the spreading. It is noted that, a pair of (α, β) values optimise the spreading simultaneously, which are marked with red solid circles in Figs. S8 and S9. For the case of the largest-degree sources in assortative networks [see Fig. S9(b)], the position of the while line in the parameter space is under that of the random sources case [see Fig. S9(a)]. This indicates that small-degree nodes should be favored more strongly when the information starts from largest-degree nodes. Interestingly, the red solid circle of optimal parameter combination is at $\alpha > 0$ in Fig. S9(a). That's to say, for the case of randomly chosen source nodes in assortative networks, large-degree nodes in network core should be preferentially contacted in the early stage, and then preferentially contact neighbors with low informed density in the periphery. For the largest-degree case in Fig. S9(b), large-degree nodes don't need to be preferentially contacted in the early stage because the sources start in the core. How different network structures influence the

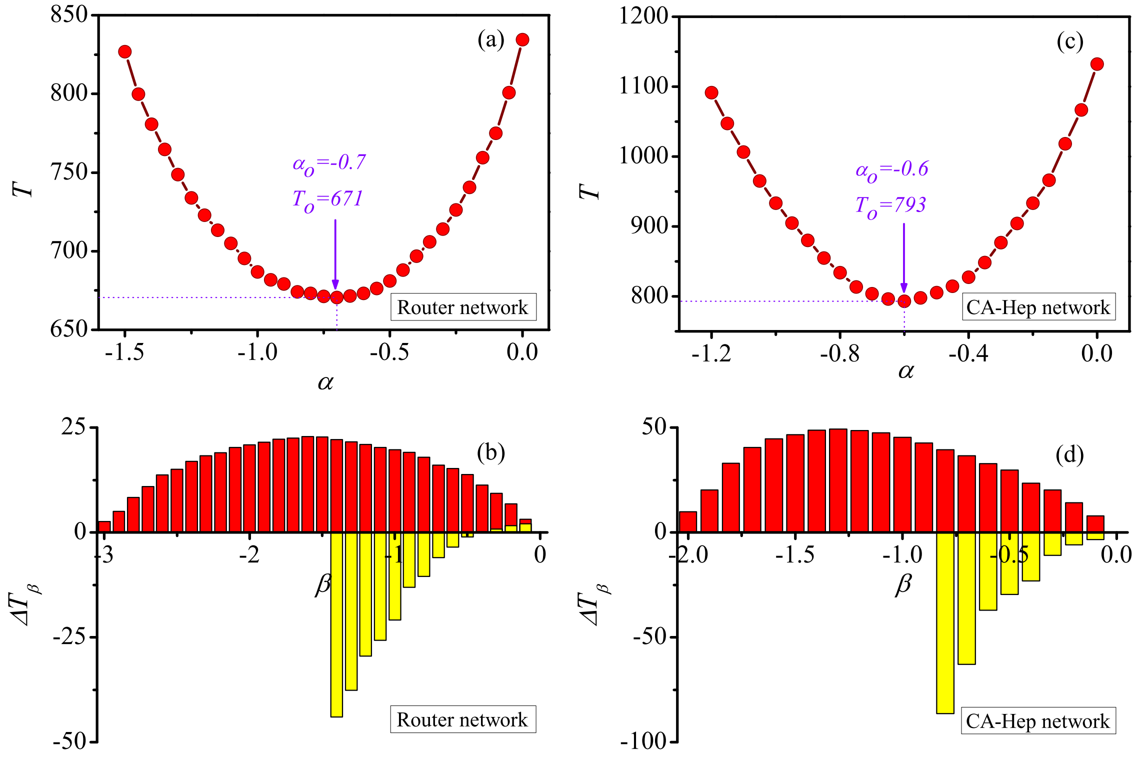

FIG. S3: **The relationship between T and α (top), and the effects of LID and GID based PCSs on the convergence time (bottom) in the real Router network [(a) and (b)] and CA-Hep network[(c) and (d)].** The correlation coefficient of the real Router network and CA-Hep network are -0.216 and 0.247, respectively. It is noted that the results for the GID case are not shown when $\beta \leqslant -1.5$ in subfigure (b) and $\beta \leqslant -0.9$ in subfigure (d), because their values exceed the minimum value of vertical coordinates. Obviously, ΔT_β monotonically increases with β.

optimal parameter combination deserve further investigation in more synthetic and real-world networks.

[1] Dorogovtsev, S. N., Goltsev, A. V. & Mendes, J. F. F. k-core organization of complex networks. *Phys. Rev. Lett.* **96**, 040601 (2006).
[2] Albert, R. & Barabási, A.-L. Statistical mechanics of complex networks. *Rev. Mod. Phys.* **74**, 47 (2002).
[3] Newman, M. E. J. *Networks: An Introduction* (Oxford University Press, Oxford, 2010).

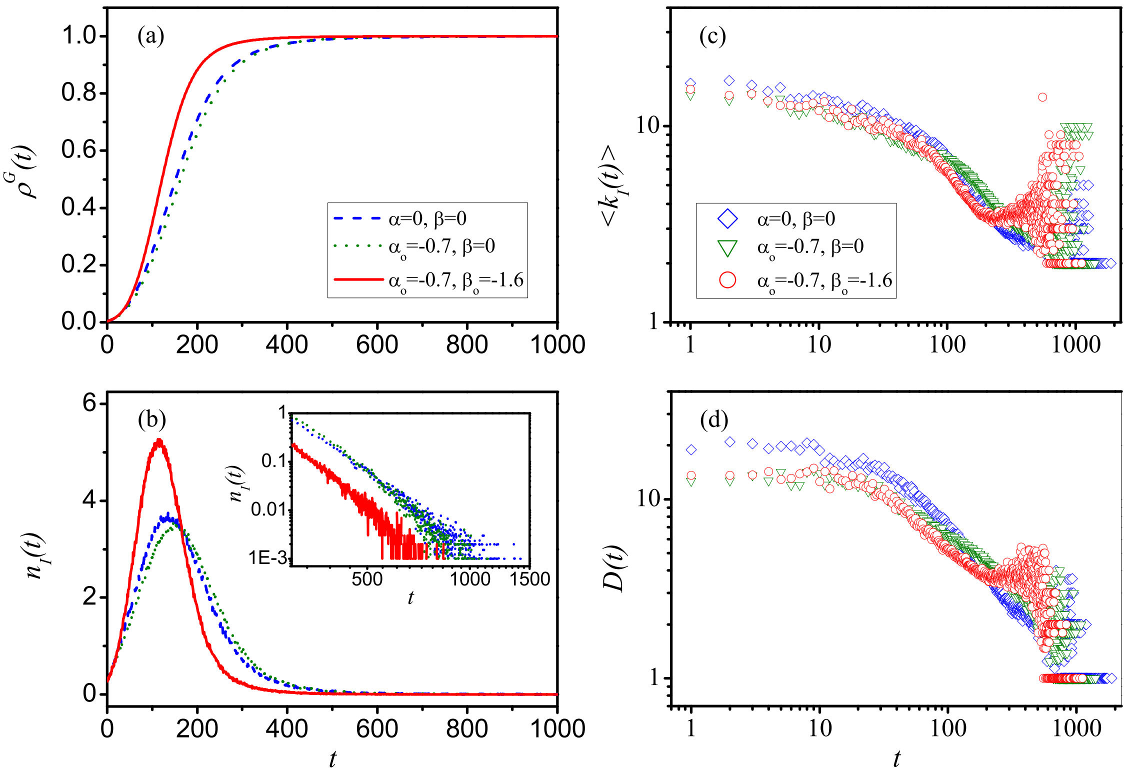

FIG. S4: **In real Router network, the effects of different contact strategies on the time evolution of information spreading.** (a) The informed density $\rho^G(t)$, and (b) the number $n_I(t)$, (c) mean degree $\langle k_I(t)\rangle$, and (d) degree diversity $D(t)$ of newly informed nodes versus t for different values of α and β. The inset of (b) shows $n_I(t)$ in the time interval $[300, 1500]$.

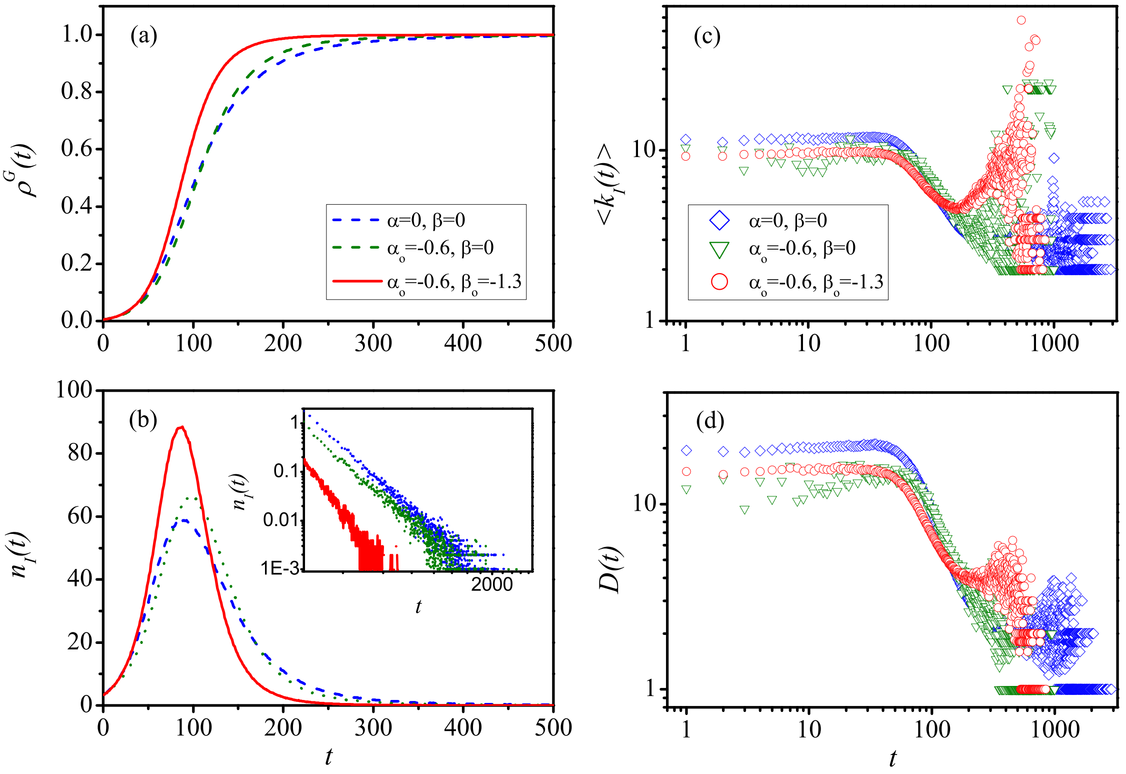

FIG. S5: **In real CA-Hep network, the effects of different contact strategies on the time evolution of information spreading.** (a) The informed density $\rho^G(t)$, and (b) the number $n_I(t)$, (c) mean degree $\langle k_I(t)\rangle$, and (d) degree diversity $D(t)$ of newly informed nodes versus t for different values of α and β. The inset of (b) shows $n_I(t)$ in the time interval $[300, 3000]$.

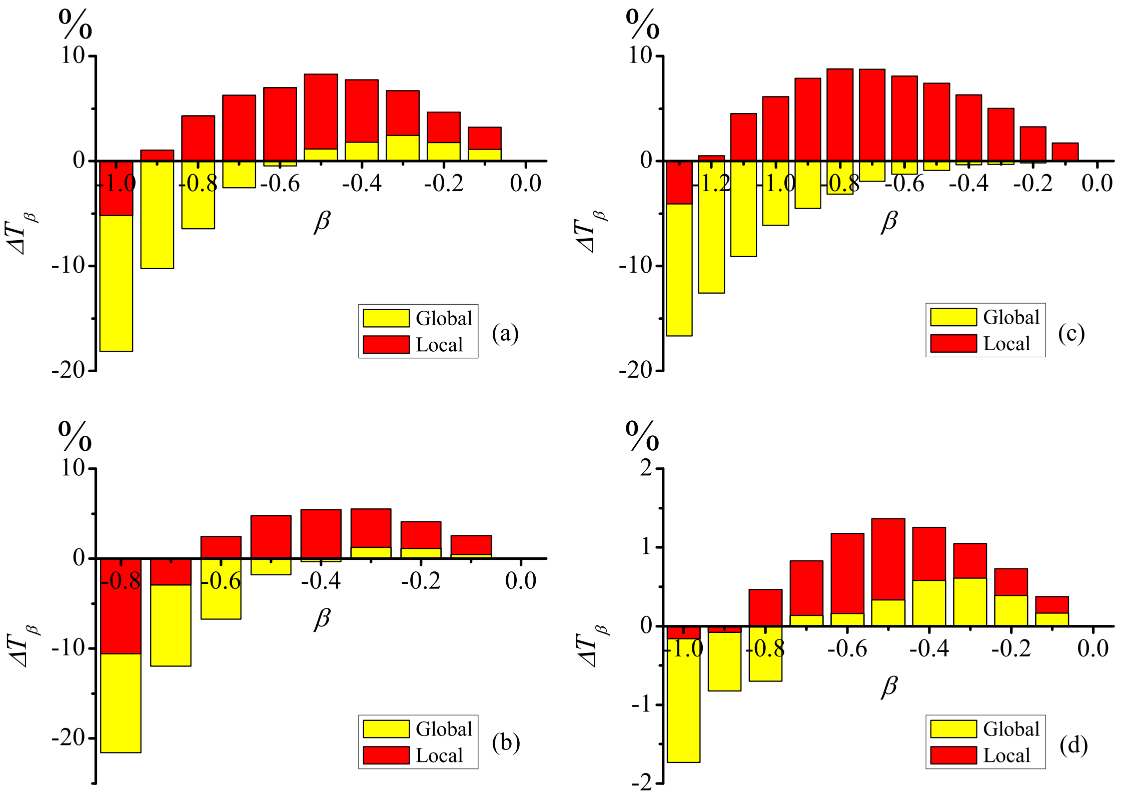

FIG. S6: **In correlated Router networks, comparison of the effects of LID and GID based PCSs on the convergence time.** The relative ratio of the convergence time ΔT_β versus β for different r values: (a) $r = 0$, $\alpha = -1.2$, (b) $r = 0.4$, $\alpha = -1.0$, (c) $r = 0.65$, $\alpha = -2.25$, and (d) $r = -0.55$, $\alpha = -1.8$, respectively.

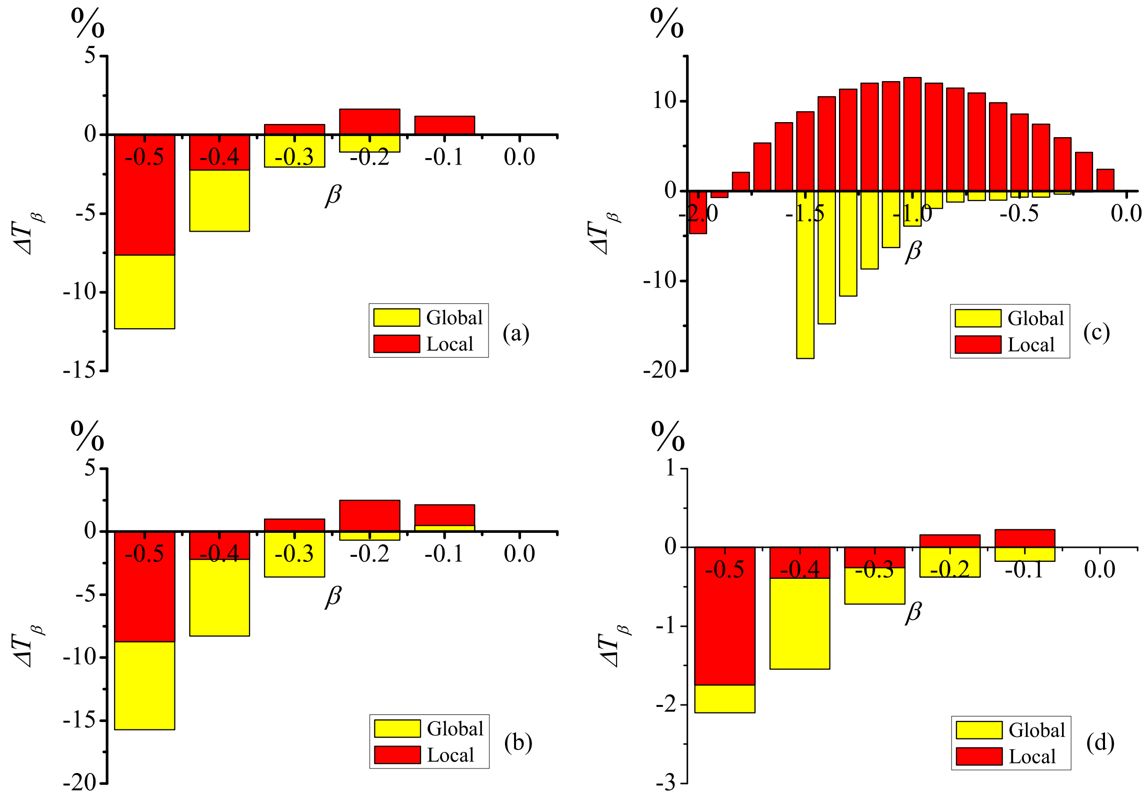

FIG. S7: **In correlated CA-Hep networks, comparison of the effects of LID and GID based PCSs on the convergence time.** The relative ratio of the convergence time ΔT_β versus β for different r values: (a) $r = 0, \alpha = -1.4$, (b) $r = 0.6$, $\alpha = -1.1$, (c) $r = 0.9$, $\alpha = -1.95$, and (d) $r = -0.65$, $\alpha = -1.9$, respectively.

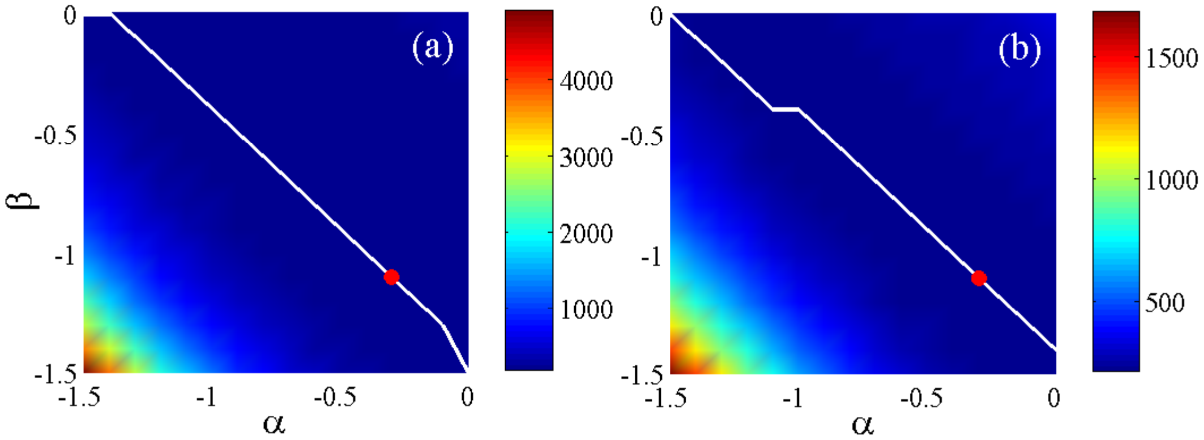

FIG. S8: **Heat maps of the convergence time in parameter space (α, β) with (a) randomly selected sources and (b) large-degree nodes greedily selected sources in uncorrelated networks.** The color indicates the convergence time. For each realization, a 5‰ of nodes are selected as seeds. The white lines correspond to the optimal β with fixed α, and the red solid circles represent the points of the minimum convergence time in parameter space (α, β). We set other parameters as $N = 10^4$, $\gamma = 3.0$, $\langle k \rangle = 8$, $\lambda = 0.1$.

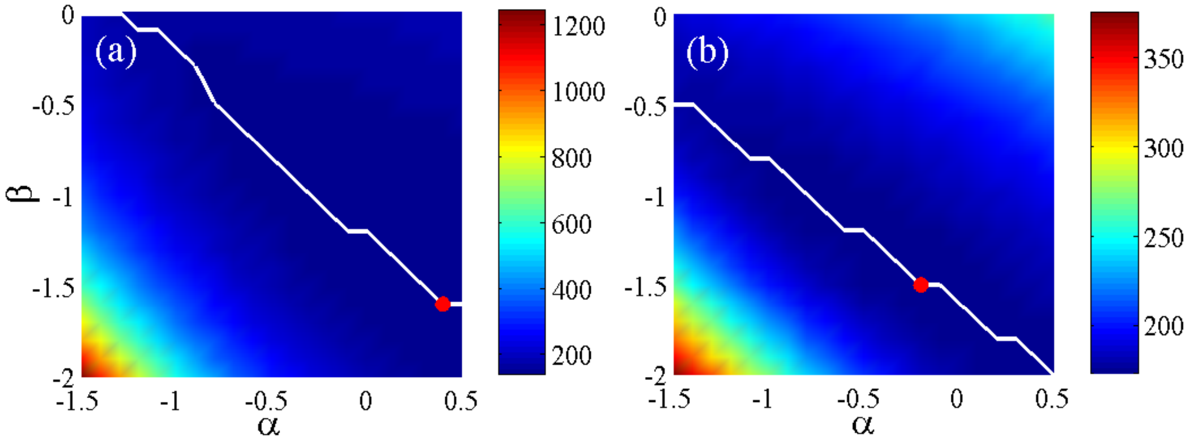

FIG. S9: **Heat maps of the convergence time in parameter space (α, β) with (a) randomly selected sources and (b) large-degree nodes greedily selected sources in assortative networks with r = 0.75.** The color indicates the convergence time. For each realization, a 5‰ of nodes are selected as seeds. The white lines correspond to the optimal β with fixed α, and the red solid circles represent the points of the minimum convergence time in parameter space (α, β). We set other parameters as $N = 10^4$, $\gamma = 3.0$, $\langle k \rangle = 8$, $\lambda = 0.1$.
